# Effect of favipiravir and an anti-inflammatory strategy for COVID-19

**DOI:** 10.1186/s13054-020-03137-5

**Published:** 2020-07-09

**Authors:** Hitoshi Yamamura, Hiroshi Matsuura, Junichiro Nakagawa, Hiroshi Fukuoka, Hisaya Domi, Satoru Chujoh

**Affiliations:** Osaka Prefectural Nakakawachi Emergency and Critical Care Center, 3-4-13 Nishiiwata, Higashiosaka, Osaka, 578-0947 Japan

**Keywords:** Favipiravir, Inflammatory, Cytokine

Favipiravir (T-705; 6-fluoro-3-hydroxy-2-pyrazinecarboxamide) is an anti-viral agent that selectively and potently inhibits the ribonucleic acid (RNA)-dependent RNA polymerase of RNA viruses [1]. In Japan, it is approved for use with novel influenza virus and is thought to be an effective drug for severe acute respiratory syndrome coronavirus 2 (SARS-CoV-2). Coronavirus disease 2019 (COVID-19) presents a complex pathology including inflammation, endothelial damage, thrombus formation, and acute respiratory failure [2–4] This syndrome requires complex treatment to reduce viral genome amounts, anti-inflammatory drugs, and anticoagulation. We attempted the cocktail treatment of favipiravir, steroid, and heparin for COVID-19. This study aimed to evaluate the effect of cocktail therapy for severe COVID-19.

This prospective, single-center study was conducted on all patients admitted to our hospital by transfer from other hospitals who required mechanical ventilation for severe COVID-19 between April 2 and 27, 2020. COVID-19 was diagnosed with real-time reverse transcriptase-polymerase chain reaction in approved laboratories from nasopharyngeal and throat swabs and with lung computed tomography. The treatment protocol was as follows: oral favipiravir (3600 mg on day 1, 1600 mg from day 2 to day 14), methylprednisolone (1000 mg for 3 days), and low molecular weight (2000 IU every 12 h) or unfractionated heparin (10,000–12,000 IU/day). Methylprednisolone administration was begun on the 5th day from initial favipiravir administration. Heparin and dexmedetomidine were administered after intubation and mechanical ventilation was started. Wilcoxon signed-rank tests were used to assess patients’ changes in paired PaO_2_/FIO_2_ (P/F) ratio, IL-6, and presepsin. All statistical analyses were performed with JMP, Version 13.0.0 (SAS Institute Inc.).

Thirteen patients with COVID-19 requiring mechanical ventilation were admitted during the study period. The treatment protocol was completed without any clinically important change in 2 patients with bronchial asthma, one with rheumatoid arthritis (early-stage steroid administration) and one with bleeding complication (without heparin). Patient characteristics and initial laboratory data are summarized in Table 1. Mean patient age was 63 (range, 46–80) years, and 69.0% were men. Only one patient required extracorporeal membrane oxygenation. Time from first symptom appearance to favipiravir administration was 8.7 (range, 4–13) days. One patient died due to disseminated intravascular coagulation (DIC) on admission that gradually progressed to multiple organ failure.

**Table 1:** Characteristics of 13 patients with COVID-19

Characteristics	Value
Age, mean (SD), years	63 (12)
Male sex, No. (%)	9 (69)
Body weight, mean (SD), kg	70 (21)
Bronchial asthma No. (%)	1 (8)
Diabetes mellitus, No. (%)	7 (54)
Hypertension, No. (%)	8 (62)
Sleep apnea syndrome, No. (%)	3 (23)
APACHE II score, median [IQR]	9 [5, 13]
PaO_2_/FIO_2_ ratio at intubation, mean (SD)	210 (73)
D-dimer on admission, median [IQR], ng/mL	1.3 [1.0, 2.8]
CRP on admission, median [IQR], mg/dL	11.9 [4.6, 19.6]
PCT on admission, median [IQR], ng/mL	0.21 [0.09, 0.54]
Presepsin on admission, median [IQR], pg/mL	440 [302, 763]
IL-6 on admission, median [IQR], pg/mL	77.5 [35.7, 161.9]
Time from first symptom appearance, mean (SD), day	
Favipiravir	8.7 (2.6)
Heparin	10.8 (3.9)
Steroid	13.2 (2.9)
Dexmedetomidine	10.7 (4.1)
Intubation	9.9 (3.3)
ECMO, No. (%)	1 (8)
Ventilator days, median [IQR], days	11 [9, 12]
Outcome, No. (%)	
Survived	12 (92)
Dead	1 (8)

*SD* standard deviation, *APACHE II* Acute Physiology and Chronic Health Evaluation II, *IQR* interquartile range, *PaO*_*2*_ arterial partial pressure of oxygen, *FIO*_*2*_ fraction of inspiratory oxygen, *CRP* C-reactive protein, *PCT* procalcitonin, *IL-6* interleukin-6, *ECMO* extracorporeal membrane oxygenation

Continuous variables were reported as median [interquartile range] (IQR). Categorical variables were reported as numbers and percentages

The time course of the clinical laboratory data is shown in Fig. 1. Day 1 indicates the first day of favipiravir administration. The P/F ratio changed very little over the first 6 days and then gradually recovered. The interleukin-6 peaked on day 4 and decreased thereafter. Presepsin also peaked on day 3, remained about the same until day 6, and then decreased.

**Fig. 1:**
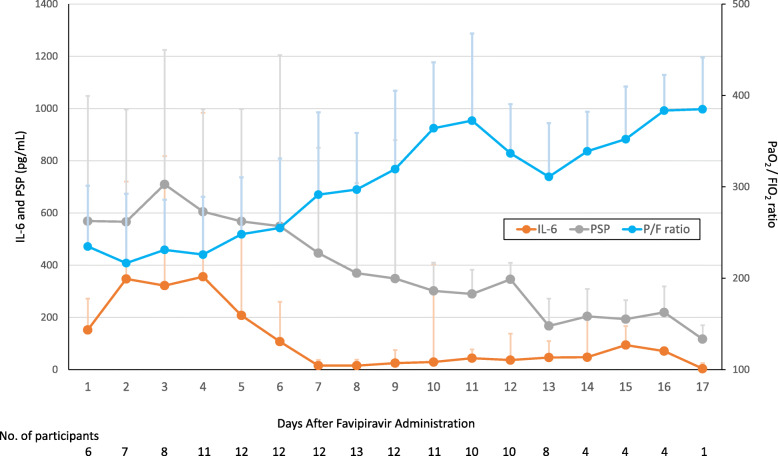
Changes of value, PaO2/FIO2, interleukin-6, and persepsin concentration in patients of SARS-CoV-2 infection. The graphs present trends in the mean (SD) values of the PaO_2_/FIO_2_ (P/F) ratio and interleukin-6 (IL-6) and presepsin (PSP) concentrations. Day 1 was the first favipiravir administration day, and administration of steroid was begun in almost all patients on day 6. The P/F ratio on day 1 was significantly lower than that on days 8–11 (*P* < .05). The IL-6 concentration peaked on day 4 and gradually decreased after that. The IL-6 on day 1 was significantly higher than that on days 7–12 (*P* < .05). The PSP on day 1 was significantly lower than that on days 7 and 11 (*P* < .05)

The clinical course of SARS-CoV-2 treatment with the cocktail in mechanically ventilated patients with COVID-19 indicated that favipiravir could partially control inflammatory mediators but could not completely control them or respiratory status. The respiratory distress of SARS-CoV-2 is thought to be due not only to direct viral action but also to chemical mediators induced by SARS-CoV-2. Inflammation and cytokine storm continued after favipiravir administration, and they could be controlled with steroid in our patients. Limitations of this study include its small sample size and performance in a single medical center. The start of favipiravir administration was delayed. Steroid therapy has adverse effects, and we did not assess long-term complications [3]. Nevertheless, the results suggested that favipiravir was of some benefit, and the findings helped inform a treatment strategy for severe COVID-19.

## Data Availability

The datasets generated and/or analyzed during the current study are not publicly available due to institutional policy but are available from the corresponding author on reasonable request.

## Abbreviations

RNA: Ribonucleic acid
COVID-19: Coronavirus disease 2019
SARS-CoV-2: Severe acute respiratory syndrome coronavirus 2
P/F: PaO_2_/FIO_2_
